# Cognitive function among older adults: ELSI-Brazil results

**DOI:** 10.11606/S1518-8787.2018052000629

**Published:** 2018-10-25

**Authors:** Erico Castro-Costa, Maria Fernanda Lima-Costa, Fabíola Bof de Andrade, Paulo Roberto Borges de Souza, Cleusa Pinheiro Ferri

**Affiliations:** I Fundação Oswaldo Cruz. Instituto René Rachou. Núcleo de Estudos em Saúde Pública e Envelhecimento. Belo Horizonte, MG, Brasil; II Fundação Oswaldo Cruz. Instituto René Rachou. Programa de Pós-Graduação em Saúde Coletiva. Belo Horizonte, MG, Brasil; III Fundação Oswaldo Cruz. Instituto de Comunicação e Informação Científica e Tecnológica em Saúde. Rio de Janeiro, RJ, Brasil; IV Universidade Federal de São Paulo. Faculdade de Medicina. Departamento de Psicobiologia. São Paulo, SP, Brasil

**Keywords:** Aged, Cognition, Memory, Socioeconomic factors, Idoso, Cognição, Memória, Fatores Socioeconômicos

## Abstract

**OBJECTIVE:**

To investigate macroregional variations in cognitive function in a national sample representative of the Brazilian population aged 50 years and older.

**METHODS:**

Data from the baseline of the Longitudinal Study of Brazilian Elderly (ELSI-Brazil), collected between 2015 and 2016, were used. Memory was measured by means of a 10-word list and executive function, by semantic verbal fluency, based on the naming of animals. Gender, age, education, and rural or urban residence were potentially confounding

**RESULTS:**

Among the 9,412 ELSI-Brazil participants, 9,085 were included in the analysis; 53.9% were women and the average age was 63.0 (0.42) years. After adjusting for potential confounding variables, average scores for memory and verbal fluency were lower in the Northeast region and higher in the Midwest and Southeast, respectively. In the South region, higher scores were found for immediate and combined memory. In all regions, older participants and those with lower schooling had worse scores for memory and verbal fluency.

**CONCLUSIONS:**

There are differences in cognitive function among older adults in the different macroregions, independent of age, gender, schooling, and rural or urban residence.

## RESUMO

**OBJETIVO:**

Investigar as variações macrorregionais da função cognitiva em amostra nacional representativa da população brasileira com 50 anos ou mais.

**MÉTODOS:**

Foram utilizados dados da linha de base do Estudo Longitudinal dos Idosos Brasileiros (ELSI-Brasil), coletados entre 2015 e 2016. A memória foi aferida por meio de lista de 10 palavras e a função executiva, pela fluência verbal semântica, baseada na nomeação de animais. Como potenciais variáveis de confusão, incluímos: sexo, idade, escolaridade e residência rural ou urbana.

**RESULTADOS:**

Entre os 9.412 participantes do ELSI-Brasil, 9.085 foram incluídos na análise; 53,9% eram mulheres e a média de idade foi 63,0 (0,42) anos. Após ajustes por potenciais variáveis de confusão, os escores médios para memória e fluência verbal foram menores na região Nordeste e maiores no Centro-Oeste e Sudeste, respectivamente. Na região Sul, foram encontrados maiores escores para memória imediata e combinada. Em todas as regiões, participantes mais velhos e com menor escolaridade apresentaram piores escores para memória e fluência verbal.

**CONCLUSÕES:**

Existem diferenças na função cognitiva entre adultos mais velhos nas distintas macrorregiões, que são independentes da idade, sexo, escolaridade e residência rural ou urbana.

## INTRODUCTION

Cognitive function is an important determinant of independence and better quality of life among older adults^1^. According to recent projections, the elderly population will triple in Brazil and will increase from 19.6 million in 2010 to 66.5 million in 2050^2^, making it the sixth largest elderly population in the world^3^.

Aging is a complex phenomenon and its impact on health conditions and functionality occurs heterogeneously among older adults. These differences are associated with genetic^4^, environmental and social determinants^5^ ^-^ ^8^, and individual characteristics of the elderly^9^ ^,^ ^10^. Previous population-based studies with national samples from several countries have shown that socio-demographic factors^11^ ^,^ ^12^ and differences between rural and urban residence areas^13^ ^,^ ^14^ are related to alterations in cognitive function among older adults, showing important cultural and geographic variations^11^ ^,^ ^12^ ^,^ ^15^ ^,^ ^16^.

Brazil is a country of continental proportions, has one of the highest levels of inequality^17^, occupies the 75^th^ position in the Human Development Index (HDI)^18^ and presents differences for this index among its macroregions^19^. There has been an increasing number of studies on the cognitive function of Brazilian older adults^20^. However, it was observed that the majority (72%) was done in the Southeast and none of these studies had a design that allowed the comparison of cognitive function among Brazilian macroregions^20^.

The Brazilian Longitudinal Study of Aging (ELSI-Brazil) used a national sample representative of individuals aged 50 years or older and allows us to investigate the cognitive function of older Brazilian adults. Thus, the objective of the present study was: 1) to investigate the cognitive function of older adults stratified by the Brazilian macroregions; 2) to compare the association between sociodemographic factors and the place of residence with cognitive function and the variations between the macroregions; 3) to investigate whether the variations in the sociodemographic structure and the place of residence observed are totally or partially responsible for the potential differences in cognitive function among macroregions.

## METHODS

### Data Source

The ELSI-Brazil is a population-based cohort study, designed to represent the Brazilian population aged 50 years or older, and with the objective of investigating the dynamics of aging in the Brazilian population and its determinants. The baseline was established between the years 2015/2016. More details can be found on the research’s homepage^a^ and in another publication^21^.

ELSI-Brazil baseline data collection included: 1) a household interview; 2) an individual interview with the participant; 3) physical measurements; 4) laboratory tests; 3) storage of blood aliquots for future analysis.

### Cognitive Function

The study adopts a conceptual framework common to other large-scale longitudinal studies of aging in the world, such as the Health and Retirement Study^21^.

This specific concept of the cognitive function module in ELSI-Brazil allows direct comparison of Brazilian results with results found in other countries, such as: China [*the China Health and Retirement Longitudinal Study* (https://g2aging.org/?section=study&studyid=4)], the United States [*the US Health and Retirement Study* (http://hrsonline. isr.umich.edu)], England [*the English Longitudinal Study of Ageing* (http://www.natcen.ac.uk/elsa)], Mexico [*Mexican Health and Aging Study*(mhasweb.org/Resources.aspx)] etc.

In the present analysis, we selected two variables that represent three well-established domains of cognitive function: memory, language and executive function. Memory was evaluated through the word list learning test, in which 10 words were read to the participants. Immediate memory was defined as the repetition of the words immediately after their reading. Late memory (delayed recall) was their repetition five minutes after presentation. The participant was kept busy filling out the questionnaire during the interval between the two tasks. Combined memory was obtained by the sum of the scores for immediate and late memory. Language and executive function were measured by the semantic verbal fluency test (animal category), in which participants were asked to say the name of as many animals as they could in a one-minute period.

### Other Variables

Also considered were the variables gender, age (50-59, 60-69, 70-79, 80 years or older), schooling (< 4, 4-7, 8-11, > 11) and place of residence (rural or urban).

### Ethical Aspects

The ELSI-Brazil Project was approved by the Ethics Commission of Fiocruz, Minas Gerais (CAAE 34649814.3.0000.5091). All participants signed separate informed consent forms for all research procedures.

### Statistical Analyses

Statistical analyzes were conducted in the Stata 14.1 program. Due to the complex design of the sample with stratification, all analyzes were done using weighting factors for each stratum, thus obtaining in the final results the contribution of each stratum according to its actual weight, and not through its participation in the sample. The use of Stata’s svy command allowed the output of robust standardized error.

To compare the characteristics of the participants among the macroregions, the analysis of variance (ANOVA) was weighted for continuous variables and the Pearson’s chi-squared test was weighted for the categorical variables.

Multivariate linear regressions with a 95% confidence interval were performed to estimate the relationship between memory (immediate, late and combined) and verbal fluency with gender, age, schooling and place of residence for each macroregion.

Finally, the average of the variables memory (immediate, late and combined) and verbal fluency was standardized separately and then simultaneously, by gender, age, schooling and place of residence, using the direct method^22^ for each macroregion.

## RESULTS

Of the 9,412 ELSI-Brazil baseline participants, 9,085 (96.5%) presented complete information for all variables and were included in this analysis. The main characteristics of the participants by region are presented in Table 1. In the total sample, the average age was 63.0 years (SE = 0.42) and the majority were female (53.9%) and had less than 11 years of schooling (76.2%). Table 2 shows the average scores and multivariate analyzes of the association between memory and verbal fluency scores stratified by macroregions. Residents in the Southeast, South and Midwest regions had the best performances, both in the memory and in the verbal fluency evaluation. Older adults in the Southern macroregion had the best performance in the variables immediate memory and combined memory, while the best performance in late memory and verbal fluency occurred among residents of the Midwest and Southeast, respectively. Older residents with low schooling had worse memory performance (immediate, late and combined) and worse verbal fluency in all macroregions. On the other hand, rural participants showed worse performance in immediate and combined memory in the Northeast and Southeast, and in late memory in the Southeast. In the Midwest, rural residents performed better in late and combined memory. Regarding verbal fluency, participants from the rural area of the South and Midwest macroregion, and women from the North and Midwest, presented worse performance of this function.

**Table 1:** Characteristics of the 9,085 sample participants. Brazilian Longitudinal Study of Aging (ELSI-Brazil), 2015-2016.

Characteristic	Total	North	Northeast	Southeast	South	Midwest	p
					
	n = 9,085	n = 713	n = 2,416	n = 3,825	n = 1,234	n = 897	
Gender: female (%)	53.9	49.9	54.6	54.5	53.6	52.1	0.934
Age – average (SE)	63.0 (0.42)	62.1 (0.67)	63.3 (0.75)	63.0 (0.72)	63.0 (0.92)	62.3 (1.1)	0.945
Education (years) (%)							< 0.0001
> 11	23.8	26.3	18.3	26.3	24.2	23.7	
8–10	11.9	15.6	8.9	12.9	12.2	11.3	
4–7	31.4	25.4	23.2	34.4	36.9	31.8	
< 4	32.8	32.7	49.5	26.4	26.7	33.2	
Place of residence: Rural (%)	15.3	17.7	30.3	6.5	21.6	5.6	0.006
Memory - mean (SE)							
Immediate	4.3 (0.05)	4.2 (0.11)	3.8 (0.08)	4.5 (0.06)	4.6 (0.07)	4.3 (0.07)	< 0.0001
Late	2.9 (0.06)	2.7 (0.15)	2.4 (0.09)	3.0 (0.07)	3.0 (0.09)	3.1 (0.13)	< 0.0001
Combined	7.3 (0.10)	7.0 (0.24)	6.2 (0.17)	7.5 (0.13)	7.7 (0.16)	7.6 (0.19)	< 0.0001
Verbal fluency - mean (SE)	12.6 (0.29)	11.4 (0.37)	11.4 (0.85)	13.2 (0.41)	12.6 (0.40)	12.9 (0.38)	0.012

All estimates were weighted by the sample parameters and sample weights of the subjects.

SE: standard error. For continuous and categorical variables, F tests were used, and the weighted chi-square, corrected for the study design, was used as a complex sample.

**Table 2:** Average scores and multivariate analyzes of the association between the cognitive function scores and the variables gender, age, schooling and place of residence of the 9,085 sample participants. Brazilian Longitudinal Study of Aging (ELSI-Brazil), 2015-2016.

Cognitive function	North		Northeast		Southeast		South		Midwest	
				
	Average	95%CI	Average	95%CI	Average	95%CI	Average	95%CI	Average	95%CI
Memory										
Immediate	4.22	3.99–4.45	3.83	3.65–4.00	4.51	4.40–4.63	4.60	4.45–4.75	4.43	4.30–4.56
Late	2.76	2.45–3.07	2.42	2.23–2.60	3.05	2.90–3.20	3.09	2.92–3.27	3.15	2.89–3.41
Combined	6.99	6.53–7.46	6.26	5.92–6.60	7.58	7.32–7.83	7.70	7.39–8.01	7.60	7.22–7.99
Verbal fluency	11.52	10.78–12.27	11.41	9.73–13.09	13.24	12.42–14.06	12.64	11.84–13.44	13.01	12.25–13.77

	**β**	**95%CI**	**β**	**95%CI**	**β**	**95%CI**	**β**	**95%CI**	**β**	**95%CI**

Immediate Memory										
Gender (F *versus* M)	0.22	-0.15–0.59	0.01	-0.10–0.13	0.07	-0.07–0.21	0.04	-0.17–0.27	0.03	-0.13–0.19
Age (≥ 75 *versus* < 75)	-1.95	-2.33– -1.57	-1.31	-1.45– -1.18	-1.20	-1.36– -1.03	-1.55	-1.90– -1.19	-1.49	-1.16– -1.36
Education (< 11 *versus* ≥ 11)	-0.96	-1.28– -0.64	-1.17	-1.36– -0.97	-1.02	-1.13– -0.91	-1.01	-1.19– -0.82	-1.07	-1.49– -0.66
Place of residence (rural *versus* urban)	-0.06	-0.30–0.16	-0.26	-0.39– -0.13	-0.51	-0.66– -0.36	-0.04	-0.32–0.24	0.01	-0.07–0.09
Late Memory										
Gender (F *versus* M)	0.09	-0.17–0.35	0.07	-0.07–0.21	0.07	-0.08–0.23	0.26	0.06–0.46	0.00	-0.44–0.44
Age (≥ 75 *versus* < 75)	-1.76	-2.23– -1.30	-1.13	-1.30– -0.96	-1.52	-1.70– -1.33	-1.54	-1.76– -1.32	-1.45	-1.70– -1.20
Education (< 11 *versus* ≥ 11)	-1.02	-1.27– -0.76	-1.22	-1.41– -1.03	-1.21	-1.35– -1.06	-1.11	-1.32– -0.89	-1.12	-1.57– -0.67
Place of residence (rural *versus* urban)	0.13	-0.25–0.53	-0.24	-0.53–0.05	-0.54	-0.77– -0.30	-0.22	-0.45–0.02	0.39	0.25–0.52
Combined Memory										
Gender (F *versus* M)	0.31	-0.28–0.90	0.08	-0.14–0.31	0.14	-0.12–0.42	0.31	-0.11–0.73	0.03	-0.55–0.61
Age (≥ 75 *versus* < 7 5)	-3.72	-4.52– -2.92	-2.44	-2.70– -2.19	-2.72	-3.04– -2.40	-3.09	-3.66– -2.53	-2.94	-3.25– -2.62
Education (< 11 *versus* ≥ 11)	-1.99	-2.51– -1.46	-2.39	-2.73– -2.05	-2.23	-2.47– -1.99	-2.12	-2.46– -1.78	-2.20	-3.06– -1.34
Place of residence (rural *versus* urban)	0.06	-0.50–0.63	-0.50	-0.86– -0.15	-1.05	-1.34– -0.76	-0.26	-0.74–0.22	0.40	0.22–0.58
Verbal Fluency										
Gender (F *versus* M)	-1.24	-2.04– -0.44	0.02	-2.07–2.13	-1.44	-3.34–0.45	-0.46	-0.96–0.03	-0.99	-1.72– -0.27
Age (≥ 75 *versus* < 75)	-1.84	-2.66– -1.03	-2.33	-4.35– -0.31	3.74	-2.23–9.72	-2.04	-2.71– -1.38	-2.09	-2.76– -1.42
Education (< 11 *versus* ≥ 11)	-1.57	-2.44– -0.70	-1.75	-4.04–0.52	-2.71	-3.53– -1.88	-3.60	-4.24– -2.97	-2.45	-4.15– -0.75
Place of residence (rural *versus* urban)	-0.20	-1.18–0.77	-1.07	-3.53–1.39	0.90	-3.97–5.78	-0.73	-1.46– -0.01	-1.10	-1.84– -0.36

F: female; M: male

All multivariate regressions were adjusted for gender, age, schooling and place of residence.

Standardized average memory (immediate, late and combined) and verbal fluency scores are arranged and compared between the macroregions in Table 3. The direct standardization of memory and verbal fluency averages by the individual effect of gender, age, schooling and place of residence and by the simultaneous effect of these four variables did not alter the pattern of difference between the regions.

**Table 3:** Standardized average scores of the cognitive function of the 9,085 sample participants. Brazilian Longitudinal Study of Aging (ELSI-Brazil), 2015-2016.

Cognitive function	Standardized by gender		Standardized by age		Standardized by schooling		Standardized by place of residence		Standardized by the 4 variables	
				
	Average Score	95%CI	Average Score	95%CI	Average Score	95%CI	Average Score	95%CI	Average Score	95%CI
Immediate memory										
North	4.24	4.00–4.47	4.03	3.85–4.20	4.11	3.91–4.31	4.23	4.03–4.43	4.08	3.92–4.24
Northeast	3.83	3.65–4.00	3.74	3.60–3.88	3.97	3.87–4.07	3.89	3.71–4.08	3.82	3.72–3.93
Southeast	4.51	4.40–4.63	4.44	4.34–4.55	4.37	4.30–4.44	4.45	4.36–4.54	4.38	4.31–4.45
South	4.60	4.45–4.74	4.50	4.32–4.68	4.46	4.35–4.57	4.62	4.50–4.74	4.50	4.38–4.61
Midwest	4.43	4.29–4.57	4.29	4.18–4.40	4.37	4.33–4.42	4.41	4.29–4.54	4.33	4.25–4.40
Late Memory										
North	2.76	2.45–3.07	2.56	2.24–2.87	2.65	2.26–3.05	2.76	2.44–3.09	2.62	2.25–2.99
Northeast	2.42	2.24–2.61	2.34	2.20–2.48	2.54	2.41–2.68	2.48	2.31–2.66	2.42	2.32–2.52
Southeast	3.05	2.91–3.20	2.97	2.86–3.08	2.89	2.80–2.99	2.98	2.86–3.11	2.90	2.81–2.99
South	3.10	2.92–3.28	2.99	2.81–3.17	2.96	2.81–3.11	3.12	2.98–3.27	3.01	2.86–3.16
Midwest	3.15	2.90–3.42	2.99	2.81–3.18	3.10	2.98–3.22	3.18	2.94–3.41	3.09	2.96–3.22
Combined Memory										
North	7.01	6.55–7.48	6.60	6.17–7.03	6.78	6.18–7.37	7.01	6.52–7.49	6.72	6.21–7.22
Northeast	6.26	5.92–6.61	6.09	5.83–6.35	6.52	6.30–6.74	6.38	6.04–6.73	6.25	6.06–6.43
Southeast	7.58	7.33–7.83	7.42	7.22–7.62	7.28	7.13–7.43	7.44	7.24–7.65	7.29	7.14–7.44
South	7.70	7.39–8.01	7.50	7.16–7.84	7.43	7.18–7.67	7.75	7.50–7.99	7.52	7.27–7.76
Midwest	7.60	7.22–7.99	7.29	7.02–7.57	7.49	7.35–7.62	7.62	7.28–7.96	7.46	7.28–7.64
Verbal fluency										
North	11.44	10.66–12.23	11.23	10.73–11.73	11.30	10.98–11.71	11.53	10.83–12.24	11.30	10.70–11.89
Northeast	11.41	9.69–13.13	11.23	9.79–12.66	11.79	9.88–13.71	11.60	9.66–13.54	11.54	9.53–13.55
Southeast	13.21	12.41–14.01	13.33	12.34–14.32	13.26	12.10–14.41	13.26	12.31–14.21	13.41	11.94–14.89
South	12.62	11.83–13.40	12.48	11.69–13.26	12.39	11.85–12.92	12.74	12.23–13.26	12.41	11.91–12.91
Midwest	12.97	12.22–13.71	12.75	12.07–13.44	12.86	12.32–13.40	12.87	12.14–13.60	12.77	12.11–13.44

The values of the averages observed and simultaneously standardized by the four variables are shown in the Figure.

**Figure:**
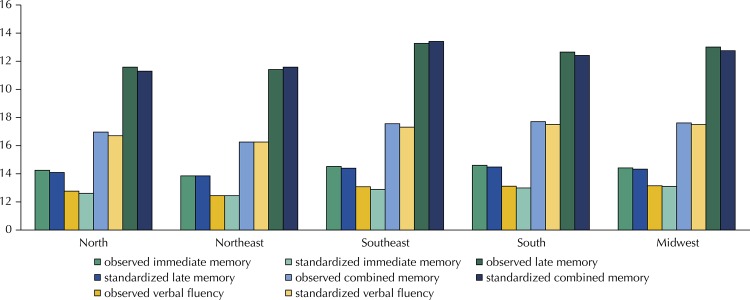
Observed and standardized average scores by gender, age, schooling and place of residence of the cognitive function of the 9,085 sample participants. Brazilian Longitudinal Study of Aging (ELSI-Brazil), 2015-2016.

## DISCUSSION

This is the first national study with a representative sample of the population aged 50 years or older that compared cognitive function in the five Brazilian macroregions, where the same tests were applied in the same period. Residents of the Southeast, South and Midwest regions presented better cognitive function in all the applied tests. However, there were variations on the ranking, depending on the ability evaluated. In all regions, there was a worsening of the three types of memory and verbal fluency correlated with the oldest age and low level of schooling, except for the Southeast macroregion, where the worsening of verbal fluency was not correlated with older age. Regarding the place of residence, we observed, inconsistently, that residents of the rural area exhibited lower scores for tests that evaluate memory and verbal fluency. However, residents of the rural area of the Midwest performed better in late memory than those in the urban area. The female gender presented the worst performance in verbal fluency tests in the North and Midwest macroregions. Finally, the standardized averages of the three types of memory and of verbal fluency by the effect of gender, age, schooling and place of residence showed that these variables did not explain all the differences found among the Brazilian macroregions.

This study has several advantages such as: 1) the use of a representative sample of Brazilian adults aged 50 and older; 2) the direct evaluation of cognitive function through validated and applied tests in the same period, which allows comparisons between the Brazilian macroregions and with other countries; 3) training and certification of the interviewers according to the protocols developed for the study; 4) quality assurance and control of data collection by conducting previous pilot studies in order to identify and correct potential problems in procedures and interviews.

However, the study also has limitations that must be considered when interpreting the results. The use of a weighted sample may underestimate the averages for cognitive function due to its design effect. However, the use of specific analyzes for weighting is likely to overcome this limitation. Although all dimensions of cognitive function were not evaluated in this study, scores of our measures (10-word list and verbal fluency test) were normally distributed with no evidence of floor or ceiling effect^23^, commonly found in low education populations.

The results of the present study are directly comparable with studies conducted in high-income countries (Health Retirement Study [HRS], English Longitudinal Study of Ageing [ELSA], the Irish Longitudinal Study on Ageing [TILDA])^11^ ^,^ ^12^ and in middle income countries, such as (Mexican Health and Aging Study [MHAS] and the Chinese Health and Retirement Longitudinal Study [CHARLS])^15^ ^,^ ^16^, in which cognitive function was assessed using the same measure for memory (a list of 10 words, with the exception of the MHAS study, which used a list of eight words) and verbal fluency (animal category semantic fluency test). ELSI-Brazil, even with a younger sample due to the inclusion of middle-aged adults, presented lower averages for all abilities evaluated (immediate, late and combined memory) when compared to the three studies in high-income countries (HRS, ELSA, TILDA)^11^ ^,^ ^12^ which used a sample of elderly individuals aged 60 years or older. Probably, this occurred due to the great difference in schooling between these samples, since this factor plays an important role in cognitive function^24^. In ELSI-Brazil, 76.2% of the sample of Brazilians aged 50 and older have less than 12 years of schooling; in HRS, ELSA and TILDA, rates are 56%, 48% and 44% for those aged 65 years and older, and 39%, 28%, 28% for those aged 57-64 years, respectively^11^ ^,^ ^12^.

Compared with studies in middle- and low-income countries, where low schooling was similar between ELSI-Brazil, MHAS and CHARLS^15^ ^,^ ^16^. It was observed that in the Mexican study, in spite of presenting an older population (adults 60 years old or older), performance in all three skills was better compared to our findings (immediate memory: 4.8 *versus* 4.3; late memory: 4.4 *versus* 2.9, and verbal fluency: 15.3 *versus* 12.6). This was because the participants in the MHAS sample were healthier than those in the ELSI-Brazil since the tests for cognitive evaluation were applied only to those who had not had a stroke or did not present depressive symptoms^16^. In addition, the memory was tested by a list of eight words, which probably influenced the results obtained with this version, overestimating the performance of the participants^16^. Regarding the Chinese study^15^, combined memory performance was worse than in ELSI-Brazil (3.3 *versus* 7.3), probably because the majority of participants were from the rural area^13^ ^,^ ^14^.

Comparisons with other Brazilian studies are limited due to the differences between the age groups and the outcomes studied. In a study with employees aged 35-74 at six universities in the Northeast, Southeast and South macroregions, memory was evaluated by a list of 10 words applied at three different occasions (immediate, late, recognition), while semantic verbal fluency was evaluated by the animal category test^25^. The averages for late memory ranged from four to eight in men and five to eight in women, while the average values for verbal fluency ranged from 12 to 21 among men and 13 to 21 among women according to participants’ schooling^25^. Like the Longitudinal Study on Adult Health (ELSA-Brazil), the present study also demonstrated that older participants with low educational level had worse performance in memory and verbal fluency. However, the scores were lower in all tests when compared to those found in ELSA-Brazil. This discrepancy probably occurred because ELSA-Brazil was conducted exclusively in the urban area (six capitals), while ELSI-Brazil was conducted both with participants living in the urban area and with those living in rural areas.

Memory and Verbal Fluency assessments, in the present study, are part of the cognitive assessment of the Consortium to Establish the Registry for Alzheimer’s Disease (CERAD)^26^, translated and validated to Brazilian Portuguese for carrying out in Brazilian elderly population^27^. Previous studies have shown that older people with lower levels of schooling and those coming from rural areas have worse cognitive performance^11^ ^-^ ^16^ ^,^ ^27^; data for the gender variable are inconsistent^11^ ^,^ ^12^ ^,^ ^15^ ^,^ ^16^ ^,^ ^2^ ^8^. Our findings replicated the associations previously demonstrated for memory and verbal fluency in other studies with population-based samples^11^ ^-^ ^16^ ^,^ ^26^ ^,^ ^27^, with the exception of results for late and combined memory, which showed that participants from rural areas in the Midwest had the best performance. This is likely to have happened because the main activity in this macroregion is farming, which possibly favors better socioeconomic conditions, which in turn may have contributed to this performance.

Finally, the results of the present study showed that the performance of adults aged 50 years or older on cognitive function tests is worse than that found in high-income countries (United States, England and Ireland) and inconsistent with that of middle-income countries (worse than that found in Mexico and better than that found in China). We observed that the association between sociodemographic, settlement factors and cognitive function had consistent variation patterns in the Brazilian macroregions. However, as these factors do not fully explain the differences in cognitive function in adults aged 50 years or older observed between regions, other contextual and cultural factors not investigated here may play a relevant role in the differences found.

## Footnotes

a Fundação Oswaldo Cruz. Brazilian Longitudinal Study of Aging. Rio de Janeiro; c2015 [cited 2017 Nov 28]. Available from: http://elsi.cpqrr.fiocruz.br
